# Are We Stepping Back? Findings From an Italian Study on Post‐Pandemic Changes in Nursing Education

**DOI:** 10.1111/inr.70027

**Published:** 2025-05-19

**Authors:** Erika Bassi, Alberto Dal Molin, Stefania Chiappinotto, Anna Brugnolli, Federica Canzan, Marco Clari, Maria Grazia De Marinis, Valerio Dimonte, Paola Ferri, Loreto Lancia, Roberto Latina, Zeno Gabriele Poli, Teresa Rea, Luisa Saiani, Federico Fonda, Alvisa Palese

**Affiliations:** ^1^ Dipartimento di Medicina Traslazionale Università del Piemonte Orientale Novara Italy; ^2^ Ospedale Maggiore della Carità Novara Italy; ^3^ Dipartimento di Medicina Università degli Studi di Udine Udine Italy; ^4^ Centro Interdipartimentale di Scienze Mediche Trento, Università degli studi di Trento Trento Italy; ^5^ Dipartimento di Diagnostica e Sanità Pubblica Università degli Studi di Verona Verona Italy; ^6^ Dipartimento di Scienze della Sanità Pubblica e Pediatriche Università di Torino Torino Italy; ^7^ Unità di Ricerca di Scienze Infermieristiche, Università Campus Bio‐Medico di Roma Roma Italy; ^8^ Dipartimento di Scienze Biomediche Metaboliche e Neuroscienze, Università di Modena e Reggio Emilia Modena Italy; ^9^ Dipartimento di Medicina Clinica Sanità Pubblica, Scienze della Vita e dell'Ambiente, Università degli Studi dell'Aquila L'Aquila Italy; ^10^ Dipartimento di Promozione della Salute Materno‐Infantile, di Medicina Interna e Specialistica di Eccellenza Università degli Studi di Palermo Palermo Italy; ^11^ Azienda Ospedaliera Universitaria Integrata di Verona Verona Italy; ^12^ Dipartimento di Sanità Pubblica Università degli Studi di Napoli Federico II Napoli Italy

**Keywords:** changes, implementation, nursing education, pandemic, recommendations, survey

## Abstract

**Aim:**

To describe (a) recommended changes implemented and their perceived effectiveness at the country level, (b) changes discontinued in the post‐pandemic era with reasons, and (c) research priorities in nursing education for the next five years.

**Background/Introduction:**

The COVID‐19 pandemic has pushed several nursing education transformations. However, no studies have documented changes still prevalent in the post‐pandemic era.

**Design:**

A national cross‐sectional study following the STROBE guidelines.

**Methods:**

All Italian nursing programs (*n* = 241) were targeted. Eight major and 21 subrecommendations indicating changes expected were included in the online survey to measure their implementation, their perceived effectiveness and status at the time of the survey, discontinuation reasons, and the research priorities. Descriptive and content analyses were used.

**Results:**

113 (45.5%) nursing programs participated reflecting the education received by >70% of Italian nursing students. All recommended changes have been implemented from 60.2% to 100% nursing programs, resulting in a perceived effectiveness from 4.29 (confidence interval [CI] 95%, 4.07–4.51) to 6.37 (CI 95%, 6.19–6.56) out of 7. A few recommendations were still applied at the time of the survey, while several were discontinued (from 4.8% to 61.9%) because, in order, of concern regarding their effectiveness, university/law dispositions, traditional methods reimplemented, technical/logistic difficulties and students’ requests. Digital solutions’ impact on nursing education was identified as a research priority.

**Conclusion:**

After the multifaced changes triggered by the pandemic, the pace of transformation of nursing education seems to have been decelerated.

**Implications for nursing:**

The potential regressive pattern that has emerged, wherein the previous model of nursing education is repristinated, calls for immediate action, which is also in line with the research priorities.

## 1 Background

Healthcare education during the COVID‐19 pandemic faced unprecedented challenges, resulting in several changes (Weine et al. 2021) relying on two main assumptions. The first assumption considers that healthcare students are in the privileged position to continue to receive education in the substantially same manner, both theoretically and practically, and to contribute to the pandemic management—as voluntary and/or paid resources (Barisone et al. 2022). In the second assumption, healthcare students have been assimilated into other higher institutions or university students where the traditional educational pathways have been interrupted and transformed into online courses (Carolan et al. 2020; Casacchia et al. 2021). Since the beginning of the pandemic, this second position prevailed across countries, with several investigations describing the changes introduced and their effects on all disciplines, including nursing. The Italian nursing education system was not different, with most nursing programs converting the traditional classroom lectures, skill labs, and clinical rotations into online teaching and learning. Many internships were paused for months, sometimes up to a year, and only some programs were maintained in person clinical rotations on a voluntary basis (Bassi et al. 2023a). In this context, a consistent body of evidence on how nursing education may be offered during the pandemic and its effects on students and their gained competencies has been produced to inform critical decisions in future disasters (Evans et al. 2023; McKay et al. 2022).

With the end of the pandemic declared by the World Health Organization on May 5, 2023 (Harris 2023), there has been limited debate on the post‐pandemic era of nursing education, with literature still concentrated on intra‐pandemic issues (Haslam 2021). From the sparse studies produced, two main trends seem to emerge: some countries and universities have restored the educational activities to the pre‐pandemic era without critically evaluating the intra‐pandemic transformations, sacrificing the extraordinary learning opportunity offered by the pandemic (Bassi et al. 2023a, 2023b); others have initiated a new course of education shaped around traditional elements (e.g., classroom) integrated with the changes implemented during the pandemic. Educational strategies aimed at enhancing flexibility and critical thinking, all increasing the preparedness of future generation (Badowski et al. 2021), as well as educational strategies embodying digital technologies in patient care (Clarke‐Darrington et al. 2023), have been newly introduced. Along with these changes, developments in cognitive sciences and healthcare systems have started to call for innovations, highlighting the need to transform education into a more complex system (Frenk et al. 2022).

Despite this call for action, a few countries or institutions have documented the transformations implemented in the post‐pandemic era; examples can be found in the field of hybrid teaching (Wang et al. 2024), in the partnership between the nursing school and the public health system sectors (Bullard et al. 2021), or in the renovated value of international programs (Gower et al. 2023). However, all these experiences regard specific interventions and institutions, whereas few studies (Bassi et al. 2023a; Bassi et al. 2023b) have documented the innovations expected in the post‐pandemic era at the country level based on the lessons learned during those challenging times. Moreover, to our best knowledge, no study has traced the innovations adopted and discontinued after the end of the pandemic. Universities, particularly their countries, are expected to not merely reintroduce pre‐established patterns of education but proactively launch a new course of education (Frenk et al. 2022).

### 1.1 Study Aim

The intent of this study was twofold: (a) to expand the knowledge available regarding the transition of nursing education in the post‐pandemic era, filling in the gaps in the literature; and (b) to contribute to the international debate on how future nursing education should be shaped by providing insights from Italy, the first Western country to be heavily affected by COVID‐19 (Aimmi et al. 2021), with dramatic effects on the society (GBD Demographics Collaborators 2024) and requiring an in‐depth revolution in healthcare science education (Lapolla & Mingoli 2020).

The specific aims were to describe (a) the changes implemented in nursing education as compared with those recommended at the national level and their perceived effectiveness; (b) the changes implemented and then discontinued at the end of the pandemic with the underlying reasons; and (c) the research priorities in the field of nursing education for the next five years.

## 2 Materials and Methods

### 2.1 Research Design

A national study named “Lessons Learned in Nursing Education (LessonsLearNED),” articulated in two phases, was conducted from 2022 to 2024 by a consortium of nine Italian universities (eight public, one private; five located in the north, two in the center, and two in the south of Italy ‐see the authors). The first phase was aimed at identifying a set of recommendations to guide Italian nursing education to move forward in the post‐pandemic era employing a qualitative descriptive design in 2022–2023 (Bassi et al. 2023a). The second phase was based on a descriptive study design according to the STrengthening the Reporting of OBservational studies in Epidemiology (Supplementary Table S1) (von Elm et al. 2007).

### 2.2 Setting and Participants

We targeted all 241 Italian nursing programs active at the time of the study (Mastrillo et al. 2024). They were offered by 48 universities with 20,059 places available in the first year, resulting in an estimation of 60,000 students over the three years of education. According to the study aims, the eligibility criteria included: (a) deans/coordinators or nurse educators (one for each nursing program, hereinafter participants) of the targeted nursing programs; (b) located in both public and private universities; and (c) willing to participate in the study. Therefore, a total of 241 participants were anticipated.

### 2.3 Data Collection Tool

The data were collected by a questionnaire including a list of recommendations on the expected changes to take forward nursing education in the post‐pandemic era (Bassi et al. 2023a, 2023b). The list of eight recommendations articulated in 21 sub‐recommendations (Supplementary Table S2) was developed by embodying the literature available and the lessons learned during the pandemic as shared by nurse educators, academicians, and students in multiple focus groups and a final consensus (Bassi et al. 2023a, 2023b).

The questionnaire asked to report the implementation (yes/no) of each sub‐recommendation; the perceived effectiveness (using a Likert scale from 1 “not at all” to 7 “greatly”); their status at the time of the survey (still in use, discontinued), and the reasons for their discontinuation (open‐ended question) when appropriate. Moreover, an open‐ended question was included asking which research priorities in the nursing education field should be considered in the post‐pandemic era up to the next five years. The questionnaire ended up requiring some demographic, professional (age, gender, education, and role), and nursing program data (number of students, nurse educators, and clinical preceptors).

The whole questionnaire was discussed by the research team in three online meetings, to achieve full agreement regarding its face and content validity (Allen et al. 2023); moreover, it was piloted by ten purposeful sampled (Patton 2014) nurse educators not involved in the final survey. The feasibility and clarity were confirmed, and no changes were suggested. Then, the online version of the questionnaire was checked and piloted to assess technicalities by involving the members of the team in order to remove potential issues in advance.

### 2.4 Data Collection Procedures

The questionnaire was implemented in the electronic platform EuSurvey (European Commission 2022), an online survey management system that allows creating and publishing survey forms made available by the European Commission. The online questionnaire access link was sent via email in January 2024 to the formal Italian nursing programs mailing list of the Permanent Conference of Health Professions Degree Classes (Palese et al. 2023). A deadline was established, and two reminders were sent via email. After one month, data collection concluded.

### 2.5 Data Analyses

Data analysis was performed using statistical software SPSS by IBM version 28 and R version 4.3 (R Core Team 2023). Quantitative continuous variables were assessed for normality using the Shapiro–Wilk test (Mishra et al. 2019). Normally distributed variables were presented with mean and standard deviation (SD), while non‐normally distributed variables with median and interquartile range (IQR) (The Jama 2020). Categorical variables were presented with relative and absolute frequencies. Likert scales were plotted according to the frequency distribution of answers and presented with mean and confidence interval (CI) 95% (Sullivan & Artino 2013). Moreover, to provide convenient data summaries, which can aid in interpreting trends across the number of discrete values of the Likert scale (Cooksey 2020), frequency tabulation was used and represented graphically. Specifically, data were sorted according to their negative‐oriented (scores 2 or 3), neutral (scores 4 out of 7), and positive‐oriented responses (scores from 5 to 7) calculating their relative frequencies and percentages.

The two open‐ended questions were analyzed by two researchers (FF and AP) and then shared with two other researchers (EB and SC), who worked independently. A fifth researcher (ADM) was consulted in case of disagreements. The reasons for discontinuation of the recommendations were checked, read carefully, and then categorized for similarities and differences by conducting a content analysis (Elo & Kyngäs 2008); similarly, research priorities were carefully read and categorized according to their similarities. All members of the research team were involved in multiple feedback to validate the findings progressively: they received, in advance, the raw data analysis performed to promote reflexivity and prepare them to give the contribution that was collected in the final stage of the study process.

### 2.6 Ethical Issues

The study was approved by the Internal Review Board of Udine University (Italy; Approval Number 144/2022). The online questionnaire displayed a full description of the study aims and procedures, including the process undertaken to identify the set of recommendations. Participants were also assured that they could withdraw their consent at any time during the study process. After having provided all details regarding the anonymity and the voluntary nature of participation, written informed consent was obtained from participants. No refusals were recorded.

## 3 Results

### 3.1 Characteristics of Participants

Overall, 113 participants out of the expected 241 (45.5%) responded, with a median of 53 years (IQR 14), mainly female (73.5%), and working in the nursing program for a median of 15 years (IQR 13). The majority had master's level (63.7%) education and were responsible for the clinical training (76.8%) in public universities (98%).

At the time of the survey, participants’ nursing programs enrolled a total of 14,123 students in the first year, and around the same in the second and third years, representing around 70.4% of the nursing students expected at the national level. Most nursing programs were located in the north of Italy (51.8%). The programs typically enrolled 216 students and were equipped with an average of four nurse educators and 100 clinical preceptors (Table 1).

**TABLE 1:** Participants and nursing programs characteristics.

Respondents	*N* = 113 (100%)
Age (years), median (IQR)	53 (14)
Gender (female), n (%)	83 (73.5)
Experience in nursing education (years), median (IQR)	15 (13)
Highest degree hold, n (%)	
Master's in nursing science	72 (63.7)
Postgraduate course (after the master's degree)	23 (20.4)
PhD	14 (12.4)
Bachelor's in nursing science	4 (3.5)
Role in nursing education, n (%)	
Responsible for the clinical traineeship	86 (76.8)
Dean of the bachelor's degree	9 (8)
Other (professor/nurse educator)	17 (15.2)
**Nursing program**	
Geographical location of the university, n (%)	***N* = 113 (100%)**
North Italy	58 (51.8)
Centre Italy	34 (30.4)
South Italy	20 (17.09)
Number of NS in the bachelor's degree, median (IQR)	
First year	90 (100)
Second year	70 (76)
Third year	65 (77)
Overall	216 (259)
Number of NEs employed in the bachelor's degree, median (IQR)	4 (7)
Number of Clinical NEs supervising the students in the clinical rotations, median (IQR)	100 (301)

Abbreviations: *N, n* = number; IQR = Interquartile Range; PhD = Doctor of Philosophy; NS = Nursing Students; NEs = Nursing Educators.

All the continuous variables were non‐normally distributed according to the Shapiro–Wilk test (*p* < 0.05).

### 3.2 Recommended Changes Implemented and Their Perceived Effectiveness

All recommended changes were implemented, in a range from in all nursing programs as “1.1 Maintaining distance learning as an opportunity to strengthen and complement classroom teaching, enhancing its complementary role to traditional learning and teaching activities” (*n* = 113; 100%) to a limited extent (“1.4 Investing in digital skills learning opportunities for students and administrative staff,” *n* = 68; 60.2%) (Table 2). However, participants perceived recommendation 1.1 as less effective, which was implemented by nearly all nursing programs (4.29 out of 7; CI 95%, 4.07–4.51), whereas “4.3 Promoting shared clinical reasoning and multidisciplinary approaches” was perceived as the most effective (6.37; CI 95%, 6.19–6.56) (Table 2).

**TABLE 2:** Moving nursing education in the post‐pandemic era: recommendations implemented, perceived effectiveness, those still in use or discontinued, and main reasons for discontinuation.

		Implemented		State of the art		
Recommendations		Yes *n* (%)	Perceived effectiveness^^^ mean (CI 95%)	Still in use *n* (%)	Discontinued *n* (%)	Main reason(s) for discontinuation ^†^
1.	***Acknowledging distance learning as a valuable complementary strategy***					
1.1	Maintaining distance learning as an opportunity to strengthen and complement classroom teaching, enhancing its complementary role to traditional learning and teaching activities.	113 (100)	4.29 (4.07–4.51)	43 (38.1)	70 (61.9)	University or law dispositions
1.2	Structuring and investing in dedicated online platforms for teaching and classroom technologies for blended approaches.	103 (91.2)	4.96 (4.68–5.24)	74 (71.8)	29 (28.2)	University or law dispositions
1.3	Investing in digital competence learning opportunities for teachers also with regard to designing and conducting online teaching activities.	91 (80.5)	4.94 (4.64–5.24)	60 (65.9)	31 (34.1)	Technical or logistic difficulties
1.4	Investing in digital skills learning opportunities for students and administrative staff.	68 (60.2)	5.24 (4.92–5.55)	50 (73.5)	18 (26.5)	Traditional methods reintroduced
1.5	Moving from a free approach, adopted as an emergency solution, to a structured approach in the degree course, where platforms, technologies, and systems are considered an integral part of the investment and learning environment.	94 (83.2)	5.21 (4.91–5.50)	73 (77.7)	21 (22.3)	Concerns on efficacy University or law dispositions Traditional methods reintroduced
1.6	Maintaining video recording of lessons to reinforce learning and ensure usability afterward or for students with special needs; identify areas where video recording can enhance the learning experience and outcomes and areas where it is unnecessary.	94 (83.2)	4.74 (4.40–5.07)	43 (45.7)	51 (54.3)	University or law dispositions Traditional methods reintroduced Technical or logistic difficulties
**2**	***Recognizing the potential role of distance learning also in laboratory activities***					
2.1	Identifying which learning activities could be offered online in the context of the workshops to anticipate the in‐presence session (e.g., briefing) or as post‐workshop reflection (e.g., debriefing) to maximize the time used in the workshops and the available resources.	92 (81.4)	4.86 (4.53–5.19)	47 (51.1)	45 (48.9)	Concerns on efficacy
2.2	Increasing the intensity of workshops by offering them in small groups (no more than six students) and of limited duration, thus making the available resources accessible and effective for all students.	77 (68.1)	6 (5.72–6.28)	62 (80.5)	15 (19.5)	Concerns on efficacy University or law dispositions
**3**.	***Rethinking clinical learning***					
3.1	Redesigning placements in terms of duration/hours and supervision models: ensure prolonged clinical experiences in the same setting as they allow for greater continuity.	92 (81.4)	5.67 (5.37–5.97)	75 (81.5)	17 (18.5)	Concerns on efficacy
3.2	Redesigning traineeships in terms of duration/hours and supervision models: propose a 1:1 supervision model, as it guarantees more effective learning.	91 (80.5)	6.08 (5.84–6.31)	83 (91.2)	8 (8.8)	Concerns on efficacy Traditional methods reintroduced
3.3	Considering new apprenticeship settings—reflecting current professional practice (e.g., outpatient clinics)—that go beyond the traditional operating units.	104 (92)	5.71 (5.48–5.95)	99 (95.2)	5 (4.8)	Concerns on efficacy
3.4	Maintaining decentralized internship experiences, including “close to home,” that can retain students, help them understand the needs of the community to which they belong, and ensure accessibility to a variety of settings and students, by spreading the presence of the nursing curriculum throughout the region/area including peripheral/remote areas.	83 (73.5)	5.83 (5.58–6.07)	78 (94)	5 (6)	Traditional methods reintroduced
**4**.	***Redefining the objectives of clinical learning***					
4.1	Focusing clinical learning processes on addressing patients' basic needs.	103 (91.2)	6.25 (6.07–6.44)	101 (98.1)	2 (1.9)	Concerns on efficacy
4.2	Focusing clinical learning processes on good infection control practices.	111 (98.2)	6.29 (6.12–6.46)	110 (99.1)	1 (0.9)	Don't know
4.3	Promoting shared clinical reasoning and multidisciplinary approaches.	103 (91.2)	6.37 (6.19–6.56)	100 (97.1)	3 (2.9)	Concerns on efficacy Traditional methods reintroduced Don't know
4.4	Promoting strategies to support students in dealing with complex placement situations, where the quality of the environment or mentoring strategies may be suboptimal due to the disruption caused by an unexpected event (e.g., a pandemic).	97 (85.8)	6 (5.74–6.26)	86 (88.7)	11 (11.3)	Traditional methods reintroduced
**5**.	***Reflecting on how to effectively integrate different learning spaces and times***					
5.1	Reviewing the planning of the nursing course of study taking into account the various learning activities offered, including virtual ones, which should be visible and effectively integrated with the planned timetable for lectures, workshops, and internships.	91 (80.5)	5.38 (5.08–5.68)	55 (60.4)	36 (39.6)	Traditional methods reintroduced
**6**.	***Pursuing inclusive and sustainable choices***					
6.1	Pursuing the digital transformation of curricula to promote proximity solutions, facilitating inclusiveness and sustainability by facilitating class/lab attendance.	92 (81.4)	5.08 (4.77–5.4)	42 (45.7)	50 (54.3)	Concerns on efficacy University or law dispositions
6.2	Encouraging the paperless approach at every stage of education, reflecting on what can be transferred digitally through the revision of course regulations.	82 (72.6)	5.45 (5.12–5.78)	65 (79.3)	17 (20.7)	University or law dispositions
**7**.	***Creating and supporting the modern student community***					
7.1	Researching and experimenting with new ways of generating an “academic community” of students in which their physical and virtual presence is facilitated.	85 (75.2)	5.68 (5.41–5.94)	69 (81.2)	16 (18.8)	University or law dispositions
**8**.	***Being ready: having a pandemic education plan***					
8.1	Developing an educational pandemic plan at the national and/or local level to harmonize decisions and provide for actions to maintain the continuity of nursing education and its quality.	77 (68.1)	5.57 (5.26–5.87)	40 (51.9)	37 (48.1)	University or law dispositions

Legend: *N, n* = number; CI = confidence Interval 95%.

^^^ Perceived effectiveness is expressed by a seven‐point Likert scale from 1 “not at all” to 7 “greatly.”

^†^ Described are the main reasons reported by participants in case of discontinued recommendations. When multiple reasons report the same frequency, without a prevalent reason over others, more reasons are indicated. Answering this question was not mandatory and relative frequencies were computed with the number of answers collected for each recommendation as denominator. In Supplementary Table 2, a detailed description of the answer is reported.

**FIGURE 1:**
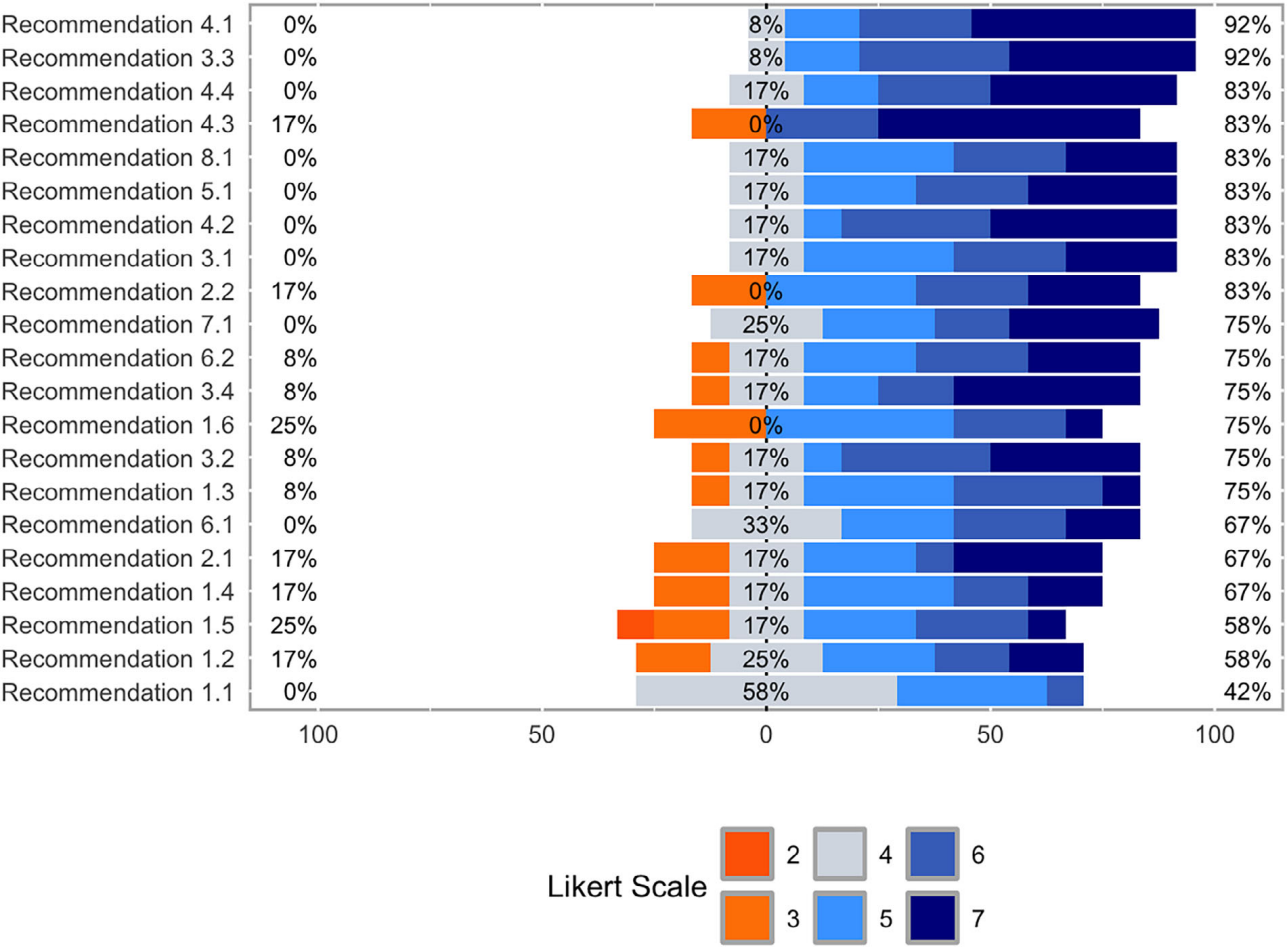
Frequency distribution of the perceived effectiveness of the recommendations according to their orientation: negative, neutral, and positive‐oriented answers. Recommendations are sorted by their perceived effectiveness distribution, expressed by a seven‐point Likert scale from 1 “not at all” to 7 “greatly.” No “not at all” responses were recorded. Legend: Recommendations *with negative‐oriented answers*: 1.2 Structuring and investing in dedicated online platforms for teaching and classroom technologies for blended approaches. 1.3 Investing in digital competence learning opportunities for teachers also with regard to designing and conducting online teaching activities. 1.4 Investing in digital skills learning opportunities for students and administrative staff. 1.5 Moving from a free approach, adopted as an emergency solution, to a structured approach in the degree course, where platforms, technologies, and systems are considered an integral part of the investment and learning environment. 1.6 Maintaining video recording of lessons to reinforce learning and ensure usability afterward or for students with special needs; identify areas where video recording can enhance the learning experience and outcomes and areas where it is unnecessary. 2.1 Identifying which learning activities could be offered online in the context of the workshops to anticipate the in‐presence session (e.g., briefing) or as a post‐workshop reflection (e.g., debriefing) in order to maximize the time used in the workshops and the available resources. 2.2 Increasing the intensity of workshops by offering them in small groups (no more than six students) and of limited duration, thus making the available resources accessible and effective for all students. 3.2 Redesigning traineeships in terms of duration/hours and supervision models: propose a 1:1 supervision model, as it guarantees more effective learning. 3.4 Maintaining decentralized internship experiences, including “close to home,” that can retain students, help them understand the needs of the community to which they belong, and ensure accessibility to a variety of settings and students, by spreading the presence of the nursing curriculum throughout the region/area including peripheral/remote areas. 4.3 Promoting shared clinical reasoning and multidisciplinary approaches. 6.2 Encourage the paperless approach at every stage of education, reflecting on what can be transferred digitally through the revision of course regulations.

Regarding the perceived effectiveness, as reported in Figure 1, from 42% to 92% recommendations received mainly positive‐oriented responses, while from 0 to 25% reported negative‐oriented responses. The most positive‐oriented (92%) recommendations were “4.1 Focusing clinical learning processes on addressing patients' basic needs” and “3.3 Considering new apprenticeship settings—reflecting current professional practice (e.g. outpatient clinics)—that go beyond the traditional units.” On the other hand, recommendations with high negative‐oriented responses (25%) were “1.5 Moving from a free approach, adopted as an emergency solution, to a structured approach in the degree course, where platforms, technologies and systems are considered an integral part of the investment and learning environment” and “1.6 Maintaining videorecording of lessons to reinforce learning and ensure usability afterwards or for students with special needs.” As indicated in Figure 1, “1.1 Maintaining distance learning as an opportunity to strengthen and complement classroom teaching, enhancing its complementary role to traditional learning and teaching activities” received the most neutral‐oriented responses (58%).

### 3.3 Changes Still in Use and Discontinued

A few recommended changes were still in use at the time of the survey (“4.2 Focusing clinical learning processes on good infection control practices”), while the remaining were discontinued in a variable range, from 61.9% (1.1. “Maintaining distance learning as an opportunity to strengthen and complement classroom teaching, enhancing its complementary role to traditional learning and teaching activities”) to 4.8% nursing programs (“3.3 Considering new apprenticeship settings—reflecting current professional practice that go beyond the traditional units”) (Table 2).

### 3.4 Reasons for Discontinuation

A total of 438 reasons (out of 488 discontinuations, 89.7%) were reported and organized into six different main categories, as follows:

1. Concerns regarding the effectiveness of the recommendation (*n* = 123; 28.1%): participants reported a lack of evidence or issues in the perceived effectiveness on learning outcomes of students as the reason for suspending some recommendations.
2. University or law dispositions (*n* = 116; 26.5%): participants reported that internal (from the rector to the dean) or external (laws or regulations) guidelines have imposed the discontinuation of certain actions (e.g., online teaching according to national rules).
3. Traditional methods reimplemented (*n* = 114; 26%): participants underlined that the patterns of education established before the pandemic were simply reintroduced, in an attempt to repristinate normality.
4. Technical or logistic difficulties (*n* = 55; 12.6%): in this case, participants mentioned that a lack of investment in digital technologies made it difficult to continue some recommendations when the pandemic was over.
5. Students’ request (*n* = 3; 0.7%) and (6) Don't know (*n* = 27; 6.2%), indicating discontinuation according to students’ claims or without a clear reason, respectively (Supplementary Table S2).

The following recommendations were discontinued (>50%) mainly due to concerns about their effectiveness: “3.1 Redesigning placements in terms of duration/hours and supervision models: ensure prolonged clinical experiences in the same setting as they allow for greater continuity” and “3.3 Considering new apprenticeship settings—reflecting current professional practice (e.g. outpatient clinics).” Universities or law disposition were reported as the reasons for discontinuation mainly regarding the “8.1 Pandemic plan” (50%), whereas the reintroduction of the pre‐pandemic education was reported as the most reasons reason for 1.3 and 1.4 recommendations concerning the investments in the digital skills of students, staff and teachers (40% and 60%, respectively). Technical difficulties were mainly cited as a cause for not maintaining video recording of the lessons (24.5%).

Three recommendations were also discontinued according to student requests (Supplementary Table S2): “1.1 Maintaining distance learning as an opportunity to strengthen and complement classroom teaching, enhancing its complementary role to traditional learning and teaching activities,” “1.4 Maintaining video‐recording of lessons to reinforce learning and ensure usability afterwards or for students with special needs,” and “6.1 Researching and trying new ways of generating an ‘academic community’ of students in which their physical and virtual presence is facilitated.”

### 3.5 Research Priorities

Overall, a total of 82 participants, out of 113 (72.6%), filled in the question by reporting 223 priorities (from 1 to 5 each), categorized into 10 main research areas, namely, (1) New digital solutions for nursing education (*n* = 62, 27.8%); (2) Emerging—new educational needs in the post‐pandemic scenario (*n* = 47, 21.1%); (3) Students’ mental health and emotion management (*n* = 22, 9.9%); (4) Community care education (20, 9.0%); (5) Nursing programs productivity (*n* = 15, 6.7%); (6) Emergency preparedness (*n* = 15, 6.7%); (7) Interdisciplinarity collaboration (12, 5.4%); (8) Tutorship and quality of education (*n* = 11, 4.9%); (9) Nursing identity development and support (*n* = 11, 4.9%); and (10) Education regarding the fundamentals of nursing care and the quality of care (*n* = 8, 3.6%).

As indicated in Table 3, the first priority was related to the new digital solutions for nursing education to investigate the effectiveness of digital literacy strategies, tools, and devices among students, teachers, and different learning settings (e.g., classroom, simulation, and clinical practice). Investigating new emerging educational needs in the post‐pandemic era was the second research priority, in terms of knowledge gaps and preferences of the new generation of students, and the role of the changes in the healthcare settings on their learning processes to inform which strategies should be enacted. The third priority was identified in the students’ mental health and emotion management, where three specifications were provided: investigating the effectiveness of strategies helping students to cope with their emotions and challenges as lived during education and in their personal life; providing knowledge on the effectiveness of strategies increasing relational skills, ethical competences, and inclusive approaches on both sides, students and teachers; and how to provide effective support from the faculty to at‐risk or frail students.

**TABLE 3:** Research priorities (*n* = 223).

Research priorities: main areas	N. of quotations	%	Research priorities: specifications	N. of quotations per each research priority	%
New digital solutions for nursing education	62	27.8	Effectiveness of interventions increasing digital literacy among students	26	41.9
			Effectiveness of interventions increasing digital literacy among teachers	19	30.6
			Effectiveness of digital solutions in classroom, simulation, and clinical practice	17	27.5
Emerging—new educational needs in the post‐pandemic scenario	47	21.1	Understanding knowledge gaps and new priorities/attitudes and preferences of students	36	76.6
			Exploring the impact of the pandemic on health services and nursing practice and changes in the acquired nursing competencies	11	23.4
Students’ mental health and emotion management	22	9.9	Effective strategies helping students to cope with emotions and promote psychological well‐being	10	45.4
			Increasing relational skills, ethical competencies, and inclusive approaches both among teachers and students	8	36.4
			Effective strategies providing academic support for frail, at‐risk nursing students	4	18.2
Community care education	20	9.0	Understanding how to implement community care education among undergraduate students (e.g., feasibility)	16	80.0
			Improving assessment competencies in chronic conditions	2	10.0
			Identifying nursing roles, responsibilities, and autonomy and distinguishing them between general and advanced community nursing care	2	10.0
Nursing programs productivity	15	6.7	Exploring the strategies to retain students and to reduce/prevent dropouts	10	66.7
			Implementing strategies to recruit students after high school	3	20.0
			Understanding the transition of newly graduated students in the post‐pandemic healthcare settings	2	13.3
Emergency preparedness	15	6.7	Increasing the effectiveness of education on risk and infection management	7	46.7
			Investigating the effectiveness of strategies increasing emergency preparedness	6	40.0
			Establishing national strategies for emergency preparedness of nursing programs	2	13.3
Interdisciplinarity collaboration	12	5.4	Understanding how to promote interdisciplinary approaches among undergraduate students	7	58.3
			Effective interventions promoting interdisciplinary education	5	41.7
Tutorship and quality of education	11	4.9	Exploring the quality of education and its impact on students' learning	7	63.6
			Understanding the roles and education of clinical educators	4	36.4
Nursing identity development and sustain	11	4.9	Defining nursing roles and autonomy	6	54.5
			Effective strategies to develop nursing identity among students	5	45.5
Education of fundamentals of nursing care and quality of care	8	3.6	Deepening fundamentals of care meaning in the context of undergraduate education	4	50.0
			Strategies to improve education awareness on the quality of nursing care	4	50.0

*n* = number.

The lowest priorities were concerned with how to develop a strong professional identity among nursing students and how to effectively teach fundamentals of nursing care and quality of care, deepening the patient‐centered care framework and improving care planning strategies.

## 4 Discussion

Several studies in nursing education have been conducted during the pandemic on required changes, their implications, and impact on students and institutions (e.g., Gordon et al. 2020; International Council of Nurses 2021). However, to our best knowledge, no studies have provided at the national level the degree of implementation of the changes recommended, their perceived effectiveness, those retained and those discontinued after the pandemic, the reasons for discontinuation, and the priorities for further research.

We involved all nursing programs resulting in a participation rate of 45.5%, in line with previous surveys involving nurses (L'Ecuyer et al. 2023); however, although the participation was limited, the nursing programs were attended by more than 70% of students in Italy.

Overall, the nursing programs involved offered education to around 200 students, equipped with four nurse educators and a network of around 100 clinical nurses working in the units to supervise students; moreover, they were mainly located in the north and the south of the country, mirroring the main features in terms of size, location, and resources of nursing programs documented in Italy as well as in international studies (Koskinen et al. 2023). Participants were educators with extensive experience in nursing education, including in the pre‐pandemic times, and held formal responsibilities, and thus were in an ideal position to provide accurate data. Their demographic and professional profile is in line with the main characteristics of educators documented previously (e.g., Bassi et al. 2023a).

### 4.1 Recommended Changes Implemented and Their Perceived Effectiveness

Almost all nursing programs have considered distance learning as a complementary strategy, with limited investments in digital skills for students and administrative staff. Digital transformation has occurred quickly, and the skills acquired could have been considered sufficient; however, given the expansion of digitalization, a clearer investment in training for teachers is also recommended, considering that around 20% of nursing programs did not implement any initiatives. Digital transformation was also implemented in skill lab settings by almost all nursing programs. Significant investment has been dedicated to rethinking clinical learning and its aims, while different learning activities for time and space (including activities delivered online) have been redesigned in almost all nursing programs. One‐third of them have implemented a strategy for community‐building and student support, thus preventing the loneliness and the sense of disruption experienced by students (Bokszczanin et al. 2023). However, not all have developed a pandemic educational plan, with the risk of leaving the system unprepared to face future disasters. Overall, nursing programs have implemented numerous recommendations, indicating that they have taken steps in many directions, demonstrating a significant transformation in nursing education. Moreover, nursing programs seem to have followed similar priorities, thus ensuring harmonization in the changes implemented. However, implementation was below 80% in 8 recommendations (e.g., “Investing in digital skills learning opportunities for students and administrative staff”) out of 21 explored: difficulties perceived locally, scarcity in resources, human resources included, and different priorities may have shaped the different degrees of implementation. In a recent country‐level study conducted in Brazil, where the transformation in nursing education has been mapped, a jeopardization of the developments achieved has emerged with a potential negative impact on education accessibility and quality (Fernandes et al. 2020). The varied pace in the implementation of the changes expected across nursing education should be considered with care.

The perceived effectiveness was generally positive‐oriented in most recommendations, suggesting their value when applied; the highest effectiveness was reported in actions focusing the clinical learning processes on patients’ basic needs and in those expanding the settings for clinical practice by including non‐traditional units, suggesting that these innovations were important. However, among the prevalent negative‐oriented recommendations (reporting scores 2 or 3 in the 25% of answers), the development of a structured approach to digitalization establishing platforms, technologies, and systems as an integral part of the investment and of the learning environment has emerged; and the maintenance of the video recording of lessons has helped to reinforce learning and ensure usability afterward for students with special needs. The negative‐oriented answers suggest that nurse educators are not convinced regarding their effectiveness, and this may be due to negative attitudes regarding digital solutions, lack of digital literacy, and the lack of data regarding their utility. Notably, evidence regarding their impact has been documented during the pandemic (e.g., Rawlings et al. 2021; Parker et al. 2023), while limited data are available in the post‐pandemic times, suggesting the need to debate the digitalization in nursing education that seems to be still controversial (Tischendorf et al. 2024). The post‐pandemic nursing education should also consider additional challenges faced by countries to best prepare the future generation of healthcare providers (Frenk et al. 2022). Future times are characterized by high uncertainty and confusion (Tognoni 2024), suggesting that a permanent debate on the future of nursing education is required at the country and international levels.

### 4.2 Changes Still in Use and Discontinued With Underlying Reasons

The implementation was continued in some actions and discontinued in others. Overall, all recommendations including the digitalization (n. 1.1, 1.6, 2.1, 6.1) have been discontinued, suggesting that the digital transition of nursing education has been interrupted or at least delayed. Despite promising evidence and the inevitable trends in the healthcare systems (Wynn et al. 2023), the perceived poor effectiveness of these solutions may have influenced the persistence in implementing the related actions; moreover, the digital attitudes of educators may have also prevented the continuation, as well as the stringent rules regarding how nursing education should be shaped and delivered in terms of hours of lessons and clinical placements, as established by the European directives (European Commission 2024; European Union 2013; European Union 2005). A rudimentary discussion about digital transformation and the curricular developments in nursing education has been produced to date (Tischendorf et al. 2024); therefore, findings of our study may reflect broader trends requiring more scientific, pedagogical, and professional debate regarding the degree of digitalization that should be implemented in nursing education.

Apart from some requests from students, which have been reported to a little extent, suggesting that the implementation of actions is led by institutions, other five main reasons have emerged, in order: issues in the effectiveness of actions—suggesting that nurse educators are concerned to reduce the preparation of future nurses; the imposition established by universities or laws, which may have established new priorities and therefore attracted resources toward them, as in the case of the pandemic plan, which was discontinued in several nursing programs; the technical and logistical challenges that may also be considered beyond the control of nurse educators, thus pending from the priorities of the universities; the reintroduction of the traditional models of education that may express the tendency to normalize the pre‐established models considering the pandemic an exceptional time; and “Don't know reasons,” suggesting that participants were not involved in the decisions regarding the suspension of certain actions. Overall, the transformation of nursing education seems to have decelerated in terms of discontinued actions: In other words, despite the call for accelerated progress and advancements (e.g., Foster & Tasnim 2020), nursing programs are hesitant about continuing the recommended transformations. Hesitancy can be triggered by the normal process of system adaptation, often reported in implementation science as that phase where the improvements are slowly introduced; on the other hand, discontinuation may also be related to issues in the sustainability of the action undertaken (Oermann et al. 2022). As reported by Frenk et al. (2022), institutions should not merely adapt their process but proactively establish new models of education and training (Frenk et al. 2022). Overall, our nursing programs seem to lack the necessary capacity, highlighting the need for specific policies and action moving forward in the education pathways in the new era (e.g., Kurtovic et al. 2021).

### 4.3 Research Priorities

The identification of research priorities is considered a useful exercise to address efforts in filling in the gaps perceived by experts in a given field. Previous exercises have involved educators in defining such priorities (Thompson, 2017; Tanner & Lindeman, 1987; Sleep et al. 1995). However, to our best knowledge, no priorities have been set in the post‐pandemic times; therefore, those that emerged represent the first attempt. Consistent with the study findings, the most prominent research priority relates to the development and evaluation of digital solutions in the nursing education context, which aligns with global trends emphasizing digital literacy (Abou Hashish & Alnajjar 2024), simulation‐based learning (Mulyadi et al. 2021; Huai et al. 2024), and the integration of advanced technologies such as augmented reality and high‐fidelity simulations. The pandemic has accelerated the adoption of digital tools; more recently, artificial intelligence (AI) has triggered additional issues not mentioned by our participants but clearly requiring investigation. Overall, international research assessing the short‐ and long‐term effectiveness of digital solutions (including AI) in nursing education on both theoretical and clinical competencies is required. A critical evaluation of its sustainability is also recommended to ensure a homogeneous growth of nursing education worldwide and prevent any division between countries where digital solutions are available or not.

Another research priority identified relates to emerging educational needs. The pandemic has reshaped healthcare environments, necessitating a reassessment of curricula to address new knowledge gaps and evolving healthcare practices (Frenk et al. 2022). This underscores the importance of flexible, adaptive educational models that can respond to more complex and rapidly changing healthcare demands. Indeed, there is a growing recognition of the need to incorporate sustainability, digital advancements, and an interdisciplinary/interprofessional approach to meet these emerging needs (Davidson 2024).

The focus on mental health and emotional management of nursing students also stands out as a pressing research priority. This aligns with the broader discourse on student well‐being, which has gained prominence during the pandemic (Rasmussen et al. 2022; Savitsky et al. 2020). Ensuring future educational strategies address the psychological and emotional challenges faced by nursing students (Viottini et al. 2024) becomes essential for fostering resilience, ethical competence, and relational skills, all of which play a fundamental role in healthcare settings (Stubin et al. 2024). In addition, designing strategies to support nursing faculties in addressing emerging needs is crucial through international studies, which may accelerate the development of evidence in different cultural contexts.

Finally, community care education and emergency preparedness are gaining importance in the post‐pandemic era. Both areas demand a more comprehensive integration into nursing curricula, ensuring that future nurses are not only prepared for acute care but are also equipped with the skills to navigate community health challenges (Italian Government 2022; Karam et al. 2021) and respond to crises effectively (Al Thobaity 2024). Paradoxical to the fact that the origin of the COVID‐19 pandemic was a zoonotic disease, no priorities have emerged regarding the strong connection between human, animal, and planetary health and their implication for education. The “One Health” approach requiring interdisciplinary collaborations to address complex issues, such as the health challenges posed by climate change and global pandemics, seems not fully considered in healthcare education (Kejriwal et al. 2024): wihile consideration should be devoted by international and national bodies to promote awareness among educators, a formal reflection of how priorities should be changed in light of the “One Health” perspectives is recommended.

### 4.4 Study Limitations

The study has several limitations. The set of recommendations to move forward the nursing education in the post‐pandemic era was designed in 2023 and used to collect data. Some recently imposed changes, for example, the expansion of the number of university places to cope with the national shortage of nurses, may have introduced additional priorities. Moreover, despite the data collection tool being subjected to content and face validity, its further use should be supported by additional validation processes. All participants were working in nursing programs, thus providing an internal perspective regarding the changes: future studies should also consider students, administrators, and the faculty/department staff to enrich the perspective. In addition, the study involved mainly public universities according to the profile of nursing programs in Italy, where the majority are offered by the public sector; consequently, findings may have limited transferability to private nursing programs.

### 4.5 Implications for Nursing and Health Policy

Overall, the transformation of Italian nursing education appears to be slowing down, as suggested by the number and the quality of the discontinued actions. If these findings reflect a slow progression in the directions suggested by expected changes or a regressive pattern toward previous models of nursing education delivery requires further investigation. However, policymakers, faculty members, and the same professional bodies should carefully consider the findings and inform actions. At the international level, replicating this research exercise may help discover similarities and differences in the expected post‐pandemic progressions and offer rich insights into global trends in nursing education across Europe, where homogeneous nursing program directives are in place, and worldwide. Nursing programs should be continuously developed, in a harmonic manner, to ensure geographical intra‐ and intercountry equality, the expected standards, and modernization, all aspects that may affect both the attractiveness of the nursing career and the competencies achieved by new graduates.

## 5 Conclusions

To our best knowledge, this is the first national study describing the changes implemented, their discontinuation and reasons in the post‐pandemic era, as well as the research priorities to address future investigations. Italy can be considered as a case study: It was affected as the first Western country at the beginning of the pandemic and, possibly, has transitioned in the post‐pandemic before others.

Overall, like other countries, Italian nursing programs have been revolutionized during the pandemic by applying several changes, suggesting a common pattern in the education transition to the post‐pandemic era. However, all recommendations, including digitalization, have been largely discontinued after the pandemic. For example, the digital transition of nursing education has been interrupted or at least delayed. Reasons are mainly external to the pedagogical debate, such as university/law dispositions, the reintroduction of traditional methods, and the technical/logistic difficulties up to don't know reasons. With the additional emerging role of AI, along with other digital tools, there is a need to open a debate among educators, the nursing profession, and relevant stakeholders regarding their value and their overall contribution to students’ learning outcomes to overcome the rudimentary discussion still present in the field of nursing education. Given the emerging leading role of digitalization, identifying the direction of nursing education development should be a priority to inform harmonized policies. Overall, moving forward, the analysis of trends from a single‐country perspective to a more international one may help establish actionable policies by educators, professional boards, and policymakers worldwide.

## Author Contributions

Conceptualization: E.B., A.D.M., and A.P. Data curation: All authors. Formal analysis: E.B., A.D.M., S.C., F.F., and A.P. Methodology: E.B., A.D.M., S.C., F.F., and A.P. Project administration: E.B., F.F., A.D.M., and A.P. Software: E.B. and F.F. Supervision: A.B., V.D., M.G.D., L.L., L.S., and F.C. Validation: All authors. Writing—original draft: E.B., A.D.M., S.C., F.F., and A.P. Writing—review and editing: All authors.

## Conflicts of interest

The authors declared no potential conflicts of interest with respect to the research, authorship, and/or publication of this article.

## Supporting information




*Supplementary Table 1*. STrengthening the Reporting of OBservational studies in Epidemiology (STROBE) Statement: cross‐sectional studies.
*Supplementary Table 2*. Recommendations: reasons for discontinuation.
