# Supplementary material for: Are We Stepping Back? Findings From an Italian Study on Post‐Pandemic Changes in Nursing Education
Source: Int Nurs Rev. 2025 May 19;72(2):e70027. doi: 10.1111/inr.70027 (PMC12086610; doi:10.1111/inr.70027)
Supplement: Supplementary file 1 — Supplementary Table 1. STrengthening the Reporting of OBservational studies in Epidemiology (STROBE) Statement: cross‐sectional studies. Supplementary Table 2. Recommendations: reasons for discontinuation. [file INR-72-0-s001.docx]

**Supplementary Table 1**. STrengthening the Reporting of OBservational studies in Epidemiology (STROBE) Statement: cross-sectional studies (von Elm et al. 2007)

|  | Item No | Recommendation | Section |
| --- | --- | --- | --- |
| **Title and abstract** | 1 | (*a*) Indicate the study’s design with a commonly used term in the title or the abstract | Title, abstract |
|  |  | (*b*) Provide in the abstract an informative and balanced summary of what was done and what was found | Abstract |
| Introduction | | |  |
| Background/rationale | 2 | Explain the scientific background and rationale for the investigation being reported | Background |
| Objectives | 3 | State specific objectives, including any prespecified hypotheses | Study aims |
| Methods | | |  |
| Study design | 4 | Present key elements of study design early in the paper | Research design |
| Setting | 5 | Describe the setting, locations, and relevant dates, including periods of recruitment, exposure, follow-up, and data collection | Setting and Participants |
| Participants | 6 | (*a*) Give the eligibility criteria, and the sources and methods of selection of participants | Setting and Participants |
| Variables | 7 | Clearly define all outcomes, exposures, predictors, potential confounders, and effect modifiers. Give diagnostic criteria, if applicable | Data collection tool |
| Data sources/ measurement | 8 | For each variable of interest, give sources of data and details of methods of assessment (measurement). Describe comparability of assessment methods if there is more than one group | Data collection tool |
| Bias | 9 | Describe any efforts to address potential sources of bias | Data collection tool, Data collection procedures |
| Study size | 10 | Explain how the study size was arrived at | Data collection procedures |
| Quantitative variables | 11 | Explain how quantitative variables were handled in the analyses. If applicable, describe which groupings were chosen and why | Data Analyses |
| Statistical methods | 12 | (*a*) Describe all statistical methods, including those used to control for confounding | Data Analyses |
|  |  | (*b*) Describe any methods used to examine subgroups and interactions | Data Analyses |
|  |  | (*c*) Explain how missing data were addressed | / |
|  |  | (*d*) If applicable, describe analytical methods taking account of sampling strategy | / |
|  |  | (*e*) Describe any sensitivity analyses | Data Analyses |
| Results | | |  |
| Participants | 13 | (a) Report numbers of individuals at each stage of study—eg numbers potentially eligible, examined for eligibility, confirmed eligible, included in the study, completing follow-up, and analysed | Characteristics of the participants |
|  |  | (b) Give reasons for non-participation at each stage | / |
|  |  | (c) Consider use of a flow diagram | / |
| Descriptive data | 14 | (a) Give characteristics of study participants (eg demographic, clinical, social) and information on exposures and potential confounders | Characteristics of the participants, Table 1 |
|  |  | (b) Indicate number of participants with missing data for each variable of interest | / |
| Outcome data | 15 | Report numbers of outcome events or summary measures | Results, Table 2 |
| Main results | 16 | (*a*) Give unadjusted estimates and, if applicable, confounder-adjusted estimates and their precision (eg, 95% confidence interval). Make clear which confounders were adjusted for and why they were included | Results, Table 2 |
|  |  | (*b*) Report category boundaries when continuous variables were categorized | Results, Table 2 |
|  |  | (*c*) If relevant, consider translating estimates of relative risk into absolute risk for a meaningful time period | / |
| Other analyses | 17 | Report other analyses done—eg analyses of subgroups and interactions, and sensitivity analyses | Results, Table 2, Figure 1 |
| Discussion | | |  |
| Key results | 18 | Summarise key results with reference to study objectives | Discussion |
| Limitations | 19 | Discuss limitations of the study, taking into account sources of potential bias or imprecision. Discuss both direction and magnitude of any potential bias | Study limitations |
| Interpretation | 20 | Give a cautious overall interpretation of results considering objectives, limitations, multiplicity of analyses, results from similar studies, and other relevant evidence | Discussion |
| Generalisability | 21 | Discuss the generalisability (external validity) of the study results | Discussion |
| Other information | | |  |
| Funding | 22 | Give the source of funding and the role of the funders for the present study and, if applicable, for the original study on which the present article is based | Funding section |

| **Supplementary Table 2**. Expected changes recommended: reasons for discontinuation. | | | | |  |  |  |
| --- | --- | --- | --- | --- | --- | --- | --- |
| **Recommendations discontinued and reasons provided (*N, 100%*)** | | ***Discontinuation reason****^^^* | | | | | |
|  |  | *Concerns on efficacy*  *n (n%)* | *University or law dispositions n (n%)* | *Traditional methods re-introduced*  *n (n%)* | *Technical or logistic difficulties*  *n (n%)* | *Students’ request*  *n (n%)* | *Don’t know*  *n (n%)* |
| **1*.*** | ***Acknowledging distance learning as a valuable complementary strategy*** |  |  |  |  |  |  |
| 1.1 | Maintain distance learning as an opportunity to strengthen and complement classroom teaching, enhancing its complementary role to traditional learning and teaching activities *(N = 61)* | 26 (37.7) | 31 (44.9) | 9 (13) | 1 (1.4) | 1 (1.4) | 1 (1.4) |
| 1.2 | Structuring and investing in dedicated online platforms for teaching and classroom technologies for blended approaches *(N = 26)* | 4 (15.4) | 9 (34.6) | 5 (19.2) | 7 (26.9) | 0 | 1 (3.8) |
| 1.3 | Investing in digital competence learning opportunities for teachers also with regard to designing and conducting online teaching activities *(N = 25)* | 3 (12) | 5 (20) | 6 (24) | 10 (40) | 0 | 1 (4) |
| 1.4 | Investing in digital skills learning opportunities for students and administrative staff *(N = 15)* | 2 (13.3) | 0 | 9 (60) | 2 (13.3) | 0 | 2 (13.3) |
| 1.5 | Move from a free approach, adopted as an emergency solution, to a structured approach in the degree course, where platforms, technologies and systems are considered an integral part of the investment and learning environment *(N = 19)* | 5 (26.3) | 5 (26.3) | 5 (26.3) | 3 (15.8) | 0 | 1 (5.3) |
| 1.6 | Maintain video recording of lessons to reinforce learning and ensure usability afterwards or for students with special needs; identify areas where video recording can enhance the learning experience and outcomes and areas where it is unnecessary *(N = 49)* | 9 (18.4) | 12 (24.5) | 12 (24.5) | 12 (24.5) | 1 (2) | 3 (6.1) |
| **2.** | ***Recognizing the potential role of distance learning also in laboratory activities*** |  |  |  |  |  |  |
| 2.1 | Identify which learning activities could be offered online in the context of the workshops to anticipate the in-presence session (e.g. briefing) or as post-workshop reflection (e.g. debriefing) in order to maximize the time used in the workshops and the available resources *(N = 45)* | 20 (44.4) | 8 (17.8) | 15 (33.3) | 1 (2.2) | 0 | 1 (2.2) |
| 2.2 | Increasing the intensity of workshops by offering them in small groups (no more than six students) and of limited duration, thus making the available resources accessible and effective for all students *(N = 15)* | 2 (13.3) | 2 (13.3) | 4 (26.7) | 6 (40) | 0 | 1 (6.7) |
| **3.** | ***Rethinking clinical learning*** |  |  |  |  |  |  |
| 3.1 | Redesigning placements in terms of duration/hours and supervision models: ensure prolonged clinical experiences in the same setting as they allow for greater continuity *(N = 16)* | 10 (62.5) | 1 (6.3) | 4 (25) | 0 | 0 | 1 (6.3) |
| 3.2 | Redesigning traineeships in terms of duration/hours and supervision models: propose 1:1 supervision model, as they guarantee more effective learning *(N = 8)* | 3 (37.6) | 1 (12.5) | 3 (37.5) | 0 | 0 | 1 (12.5) |
| 3.3 | Consider new apprenticeship settings - reflecting current professional practice (e.g. outpatient clinics) - that go beyond the traditional operating units *(N = 5)* | 3 (60) | 0 | 1 (20) | 0 | 0 | 1 (20) |
| 3.4 | Maintain decentralized internship experiences, including ‘close to home’, that can retain students, help them understand the needs of the community to which they belong and, ensure accessibility to a variety of settings and students, by spreading the presence of the nursing curriculum throughout the region/area including peripheral/remote areas *(N = 5)* | 1 (20) | 0 | 2 (40) | 1 (20) | 0 | 1 (20) |
| **4.** | ***Redefining the objectives of clinical learning*** |  |  |  |  |  |  |
| 4.1 | Focusing clinical learning processes on addressing patients' basic needs *(N = 2)* | 1 (50) | 0 | 0 | 0 | 0 | 1 (50) |
| 4.2 | Focusing clinical learning processes on good infection control practices *(N = 1)* | 0 | 0 | 0 | 0 | 0 | 1 (50) |
| 4.3 | Promoting shared clinical reasoning and multidisciplinary approaches *(N = 3)* | 1 (33.3) | 0 | 1 (33.3) | 0 | 0 | 1 (33.3) |
| 4.4 | Promote strategies to support students in dealing with complex placement situations, where the quality of the environment or mentoring strategies may be sub-optimal due to the disruption caused by an unexpected event (e.g. a pandemic) *(N = 9)* | 1 (11.1) | 0 | 4 (44.4) | 2 (22.2) | 0 | 2 (22.2) |
| **5.** | ***Reflecting on how to effectively integrate different learning spaces and times*** |  |  |  |  |  |  |
| 5.1 | Review the planning of the nursing course of study taking into account the various learning activities offered, including virtual ones, which should be visible and effectively integrated with the planned timetable for lectures, workshops and internships *(N = 36)* | 8 (22.2) | 9 (25) | 14 (38.9) | 4 (11.1) | 0 | 1 (2.8) |
| **6.** | ***Pursuing inclusive and sustainable choices*** |  |  |  |  |  |  |
| 6.1 | Pursuing the digital transformation of curricula to promote proximity solutions, facilitating inclusiveness and sustainability by facilitating class/lab attendance *(N = 49)* | 15 (30.6) | 15 (30.6) | 13 (26.5) | 5 (10.2) | 0 | 1 (2) |
| 6.2 | Encourage the paper-less approach at every stage of education, reflecting on what can be transferred digitally through the revision of course regulations *(N = 16)* | 3 (18.8) | 6 (37.5) | 4 (25) | 1 (6.3) | 0 | 2 (12.5) |
| **7.** | ***Creating and supporting the modern student community*** |  |  |  |  |  |  |
| 7.1 | Research and experiment with new ways of generating an ‘academic community’ of students in which their physical and virtual presence is facilitated *(N = 15)* | 4 (26.7) | 7 (46.7) | 1 (6.7) | 0 | 1 (6.7) | 2 (13.3) |
| **8.** | ***Being ready: having a pandemic education plan*** |  |  |  |  |  |  |
| 8.1 | Develop an educational pandemic plan at the national and/or local level to harmonize decisions and provide for actions to maintain the continuity of nursing education and its quality *(N = 10)* | 2 (20) | 5 (50) | 2 (20) | 0 | 0 | 2 (20) |

**Legend**: *N,n* = number.

^^^ Reasons reported (when reported) by respondents who discontinued recommendations. Answering this question was not mandatory in the questionnaire and relative frequencies were computed with the number of answers collected for each recommendation as denominator (*N*).

**References**

von Elm, E. et al. (2007) The Strengthening the Reporting of Observational Studies in Epidemiology (STROBE) statement: guidelines for reporting observational studies. *Annals of Internal Medicine* **147** (8)**,** 573-577. <https://doi.org/10.7326/0003-4819-147-8-200710160-00010>
